# Life-cycle assessment of three biorefinery pathways across different generations

**DOI:** 10.1038/s41598-025-96474-w

**Published:** 2025-04-16

**Authors:** Nimish Khandelwal, Sakshi Kumari, Siddharth Poduval, Shishir Kumar Behera, Ashutosh Kumar, Vidyadhar V. Gedam

**Affiliations:** 1 Industrial Ecology Research Group, School of Chemical Engineering, Vellore Institute of Technology, Vellore, Tamil Nadu 632 014 India; 2 Department of Energy and Environment, Thapar Institute of Engineering and Technology, Patiala, Punjab 147 004 India; 3 Sustainability Management Area, Indian Institute of Management (IIM), Mumbai, Maharashtra 400 087 India

**Keywords:** Lignocellulosic biomass, Microalgae, Biomass conversion, Hydrothermal liquefaction, Renewable diesel, Palm fatty acid distillate, Environmental impact, Energy and society, Sustainability

## Abstract

This study evaluated the ecological impacts of three generations of biorefineries using an integrated circularity-life cycle assessment (LCA) approach. In this context, LCA of three distinct biorefinery scenarios was conducted utilising the Greenhouse Gases, Regulated Emissions, and Energy use in Transportation (GREET^®^) model to compare their environmental impacts. Two third-generation scenarios, i.e., algae hydrothermal liquefaction (HTL)—pathway I and combined algae processing (CAP)—pathway II, and one second-generation scenario, i.e., palm fatty acid distillation (PFAD)—pathway III, were considered by following a well-to-wheel system framework. Pathway I and II exhibited very low emissions with negative net emissions in pathway I. On the contrary, pathway III had the highest emissions. Based on the results obtained from this study, exploitation of marine biomass (in the third-generation) might offer a sustainable solution to global energy demands with emerging green technologies, such as hydrothermal treatment, advanced fermentation, and green solvents that are essential for improving marine biorefineries. Policies must be formulated by setting standards prioritising renewable energy, resource efficiency, and waste reduction that are important in shaping the sustainability and efficiency of biorefineries across the globe.

**Supplementary Information:**

The online version contains supplementary material available at 10.1038/s41598-025-96474-w.

## Introduction

Biofuels help cut emissions and boost sustainability by converting non-edible biomass and waste into fuels and materials through efficient, economically viable biorefineries^1–4^. Biorefineries serve as a sustainable alternative to traditional refineries by focusing on conversion of biomass into a diverse range of value-added products^2^. Biorefineries leverage advanced processes such as enzymatic reactions and microbial transformations to overcome challenges associated with technical efficiency and economic feasibility in the bioeconomy^4^.

Biorefineries are categorised into three generations based on the type of feedstock used and the technological advancements involved. Understanding the distinctions between these generations is key to advancing sustainable and economically viable bio-based industries. First-generation biorefineries use food crops^5^, second-generation biorefineries utilise non-food lignocellulosic biomass^6^, and third-generation biorefineries leverage algae and microbial systems to convert CO_2_ into valuable products^7^. While first- and second-generations have their advantages and challenges, the progression towards third-generation biorefineries represents a significant step towards a sustainable and carbon-neutral economy.

The economic feasibility, technological efficiency, and sustainability of biorefineries are confronted with numerous challenges^2,4,8–10^. Since many biorefinery products are not yet commercially viable and their production techniques are immature, economic feasibility is still a major concern^2,4^. Efficient lignin depolymerisation and the requirement for improved catalysts and procedures are the examples of technical obstacles. The necessity for integral raw material utilisation and efficient waste stream valuation present sustainability issues^4,8^. Furthermore, managing bio-waste presents major operational issues, particularly in underdeveloped countries^9^. While novel strategies such as employing raw wastewater in microalgae-based biorefineries are critical to increase the economic viability, advancements in fermentation and enzyme technology, and the use of microorganisms are required for improving the efficiency of processes^4,10^.

Biorefineries face multifaceted challenges, including managerial, organisational, supply chain, and policy issues^10–13^. Managerial and organisational challenges, particularly in pilot and demonstration plants, can delay the development due to unclear roles, lack of specific competencies, and evolving objectives^11^. Transitioning to a bioeconomy requires overcoming inertia, strengthening actor networks, and improving policy coordination^12^. Supply chain and logistical issues, such as centralised nature of biorefineries and biomass collection economics are identified as the barriers^10,13^. Additionally, contradictory policy instruments and a lack of coordination at the government level can hinder biorefinery development. Policy integration and alignment are necessary to stimulate disruptive innovations and build sustainable value chains.

Life cycle assessment (LCA)—a comprehensive approach to sustainability analysis—is used to evaluate the environmental impacts of a product throughout its entire life cycle, starting from raw material extraction to its disposal^14–19^. LCA methodologies have progressed with distinctions between attributional and consequential LCA, advancements in database, and development of hybrid approacheswith challenges, such as lack of harmonisation of existing data^16–18^. The evolution of LCA into a life cycle sustainability assessment (LCSA) incorporates environmental, economic, and social dimensions to support decision-making across various fields^19^.

**Fig. 1:**
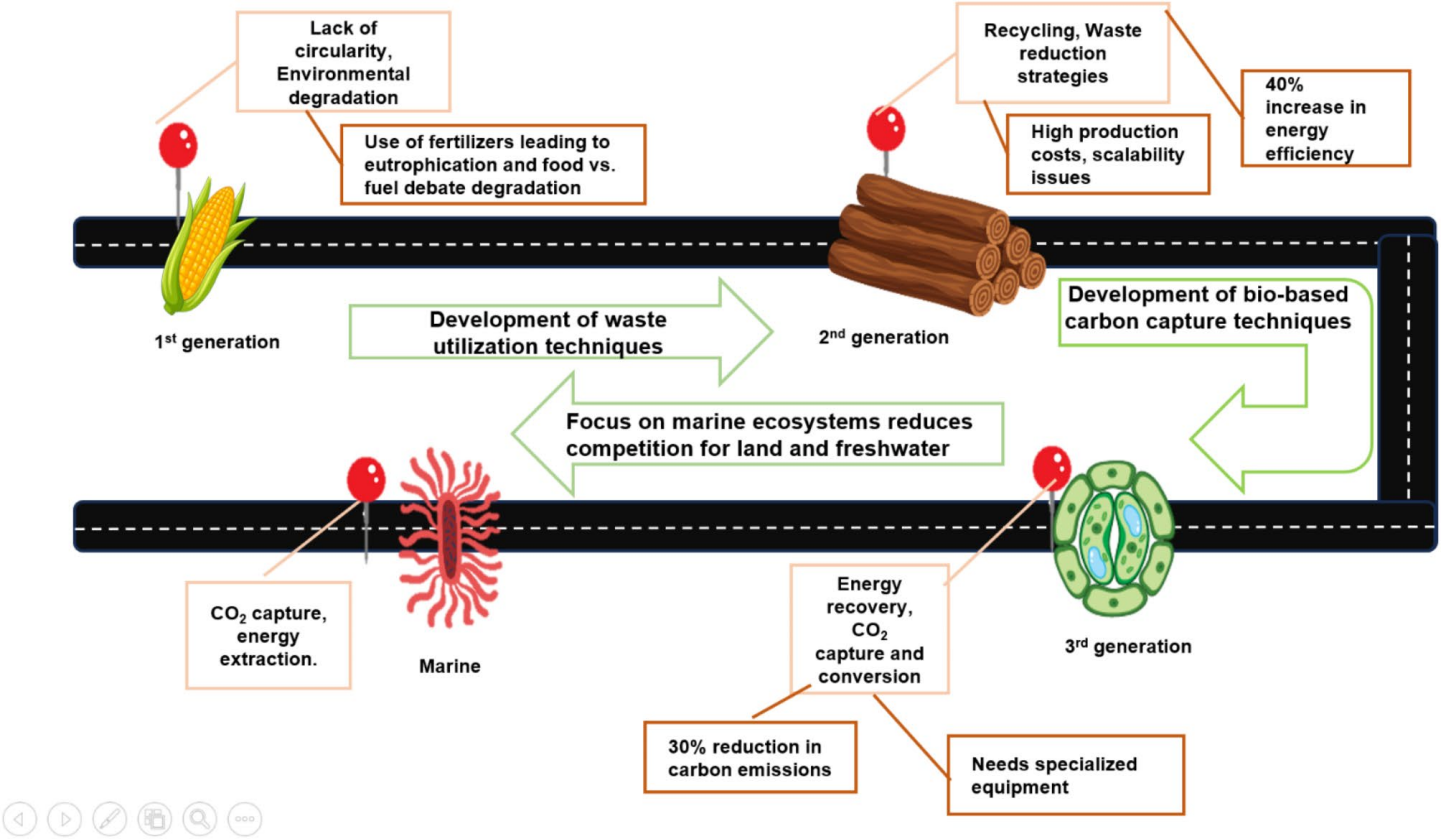
A visual overview of the development of three generations of biorefineries.

LCA plays a pivotal role in evaluating the environmental impact and sustainability of biorefineries across their generational evolution. Comparative LCA studies have highlighted the shifting paradigms from first-generation biorefineries reliance on crop-based feedstocks^14,20^ to third-generation facilities utilising innovative sources like algae and CO_2_^21,22^. These studies underscore the significant environmental benefits, such as reduced global warming potential and enhanced resource efficiency, while identifying challenges like feedstock optimisation, technological integration, and economic feasibility (Fig. 1). By analysing feedstock diversity, regional contexts, and technological advancements, methodological gaps, such as inconsistent system boundaries^23^, lack of uncertainty analysis, and incomplete land-use assessments^24^, underscore the need for standardised LCA approaches to fully realise the environmental and economic potential of biorefineries.

The evolution of biorefineries has been marked by significant improvements in environmental performance with third-generation pathways emerging as the most promising for sustainable fuel production. Compared to their predecessors, these advanced systems exhibit superior resource efficiency, reduced carbon footprints, and in some cases, net-negative emissions (Table 1). While LCA methodologies provided valuable insights into the environmental impacts of biorefineries, their application to different generational pathways remained complex due to variations in feedstock availability, technological maturity, and system boundaries. Comparative studies have underscored the potential of third-generation biorefineries in mitigating greenhouse gas (GHG) emissions and optimising resource efficiency, yet, challenges persist in harmonising LCA frameworks for a more standardised assessment^16–18^. Additionally, integrating consequential LCA approaches with emerging sustainability metrics can provide a more holistic perspective on the trade-offs and synergies across biorefinery generations^19^. Addressing these methodological deficiencies is essential for accurately quantifying the environmental benefits and informing policy decisions to support the transition towards sustainable biofuel production. The findings of this study build on these advancements by providing an LCA of selected second and third-generation biorefinery pathways, offering a nuanced comparison of their environmental trade-offs.

**Table 1:** Comparative impact assessment of biorefinery generations. *Source*: ^14,15,25–28^.

Impact types	Metrics	First-generation	Second-generation	Third-generation
Environmental impact	GHG emissions (kg-CO_2_-eq./mmBTU product)	140–150/mmBTU bioethanol	55–65/mmBTU biodiesel	−10 to 5/mmBTU biodiesel
	Energy consumption (MJ/mmBTU product)	2–3/mmBTU bioethanol	190–200/mmBTU biodiesel	2500–2600/mmBTU biodiesel
	Water requirement (L)	39,000–40,500	24,000–25,000	650–700
	Land use (ha)	5–6	0.5–0.7	0.2–0.4
Economic impact	Production cost ($/L)	2.9–3.1	2.8–3.0	2.7–2.9
	Market price ($/L)	3.0–3.1	2.8–2.9	2.5–2.7
Social impact	Job creation (number of jobs)	Moderate (agriculture)	High (specialised processing jobs)	High (research and technology jobs)
	Community development programs	Limited	Growing	Extensive, especially in rural areas
Efficiency metrics	Biomass conversion efficiency (%)	30–40	50–60	60–80
	Production yield (kg/ton of biomass)	200–250	300–350	400–500
Sustainability indicators	Renewable energy use (%)	20–30	40–50	60–80
	Waste reduction (%)	30–40	50–60	70–90
	Recyclability of by-products (%)	40–50	60–70	80–90

The primary objectives of this research paper are: (i) to provide a forward-looking introspection of the evolution of biorefineries while addressing the challenges and opportunities faced by different generations; (ii) to present a comprehensive LCA of three pilot/industrial-scale biorefinery scenarios (i.e., one second- and two third-generation pathways) for the comparison of their environmental impact in terms of GHG emissions, energy consumption, and water requirement. Moreover, the quantification of net water production in third-generation biorefineries has also been explored, underscoreing their potential sustainability advantages.

## Methodology

The GREET^®^ (Greenhouse Gases, Regulated Emissions and Energy Use in Transportation) model (version 2023)^27^, developed by the Argonne National Laboratory, was employed to simulate the energy consumption, GHG emissions, and water requirements involved in the production of 1mmBTU renewable diesel by utilising algal biomass and crude palm oil as feedstocks.

The GREET^®^ model applies a modular LCA framework to evaluate environmental impacts across the entire fuel production chain, incorporating feedstock cultivation, processing, transportation, and combustion. The model integrates matrix-based calculations and Monte Carlo simulations, refining emissions and energy estimates using real-world data sources. In this study, the GREET^®^ model was modified to reflect actual process parameters and recent experimental data, ensuring greater accuracy and representativeness of results.

**Fig. 2:**
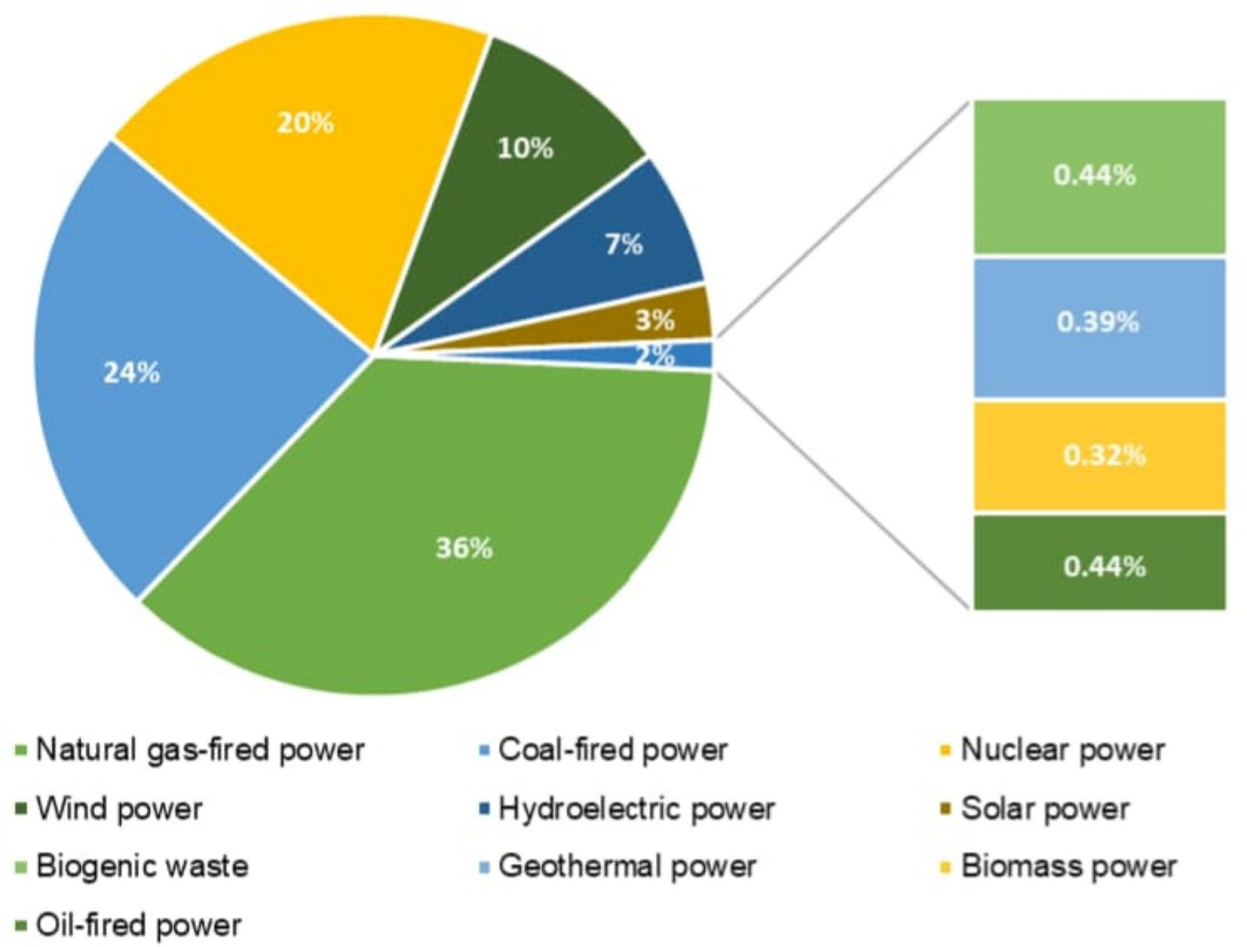
Grid electricity generation mix considered for LCA. (*Source*: GREET^®^ 2023)

**Fig. 3:**
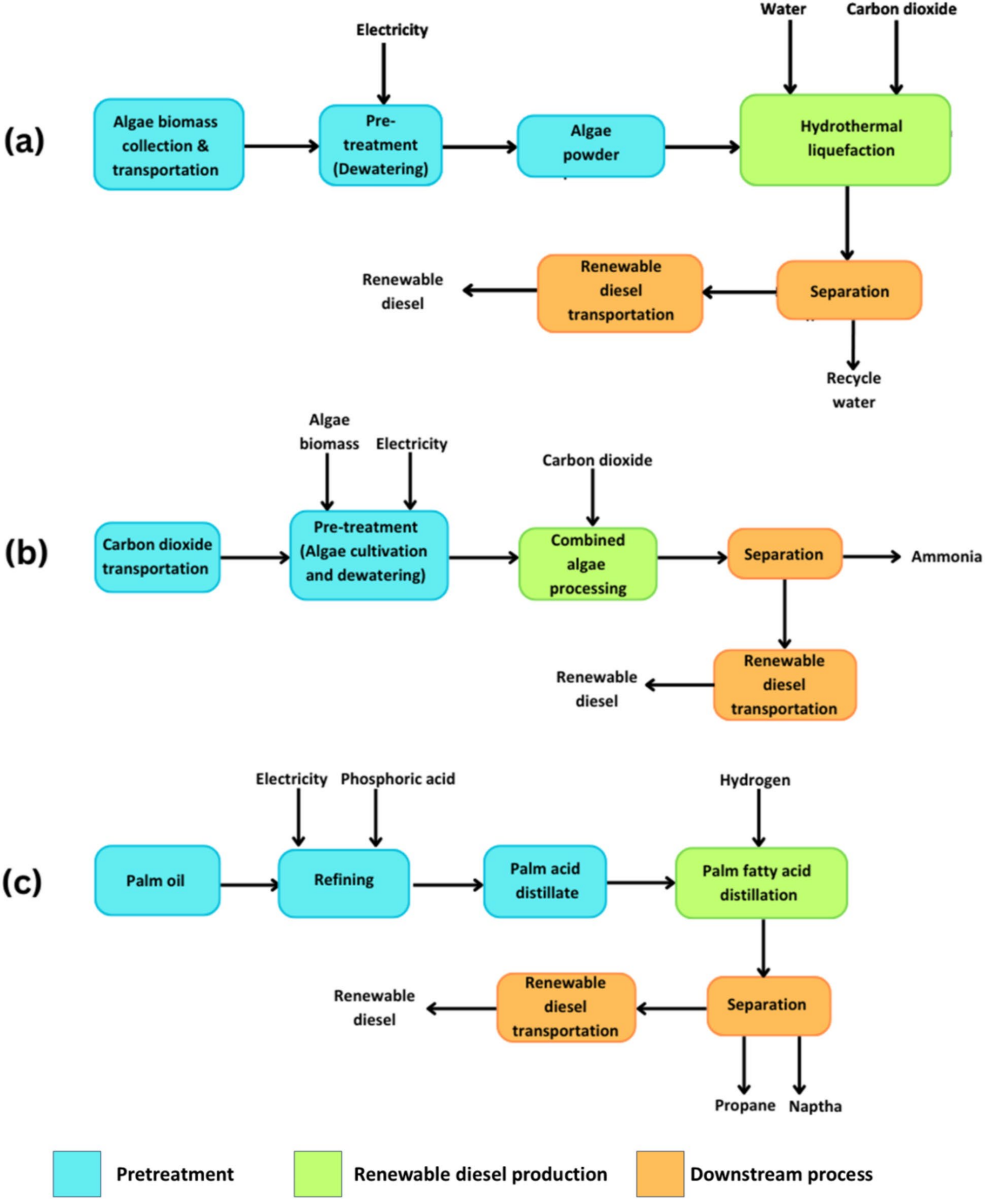
(**a**) Algae hydrothermal liquefaction, (**b**) Combined algae processing, and (**c**) Palm fatty acid distillation to produce renewable diesel. (*Source*: GREET^®^ 2023)

A well-to-wheel (WTW) system boundary has been adopted for all pathways to ensure that emissions and energy consumption across feedstock cultivation, fuel production, and transport tare accounted for. The study evaluates three scenarios for renewable diesel production: (a) Algae hydrothermal liquefaction (HTL) (Pathway-I), (b) Combined algae processing (CAP) (Pathway-II), (c) Palm fatty acid distillation (PFAD) (Pathway-III). The US electricity grid mix was selected as the default energy source for these pathways. This decision was based on the availability of comprehensive emissions data in GREET^®^, ensuring consistency across scenarios. The grid mix includes oil, coal, natural gas, biomass, wind, solar, biogenic waste, hydroelectricity, and nuclear power (Figs. 2 and 3). While palm oil production does not occur in the US, the GREET^®^ model allows for the adjustment in feedstock-specific parameters, and accordingly, the model has been modified to reflect the realistic regional energy use.

The selection of HTL, CAP, and PFAD is based on their technological significance and potential for large-scale deployment. HTL, a third-generation biorefinery technology, converts wet algal biomass into bio-crude without energy-intensive drying and is currently under development at the pilot scale^15^. CAP integrates biochemical and thermochemical processes, utilizing CO_2_ as a feedstock, but remains in the pilot stage due to cost and efficiency challenges^29^. PFAD-based renewable diesel production, a second-generation pathway, has reached the industrial stage, particularly in Southeast Asia and Europe, though it raises concerns over land use and sustainability^30^. Analysing these pathways provides a comprehensive assessment of their environmental feasibility and scalability for sustainable biofuel production.

**Fig. 4:**
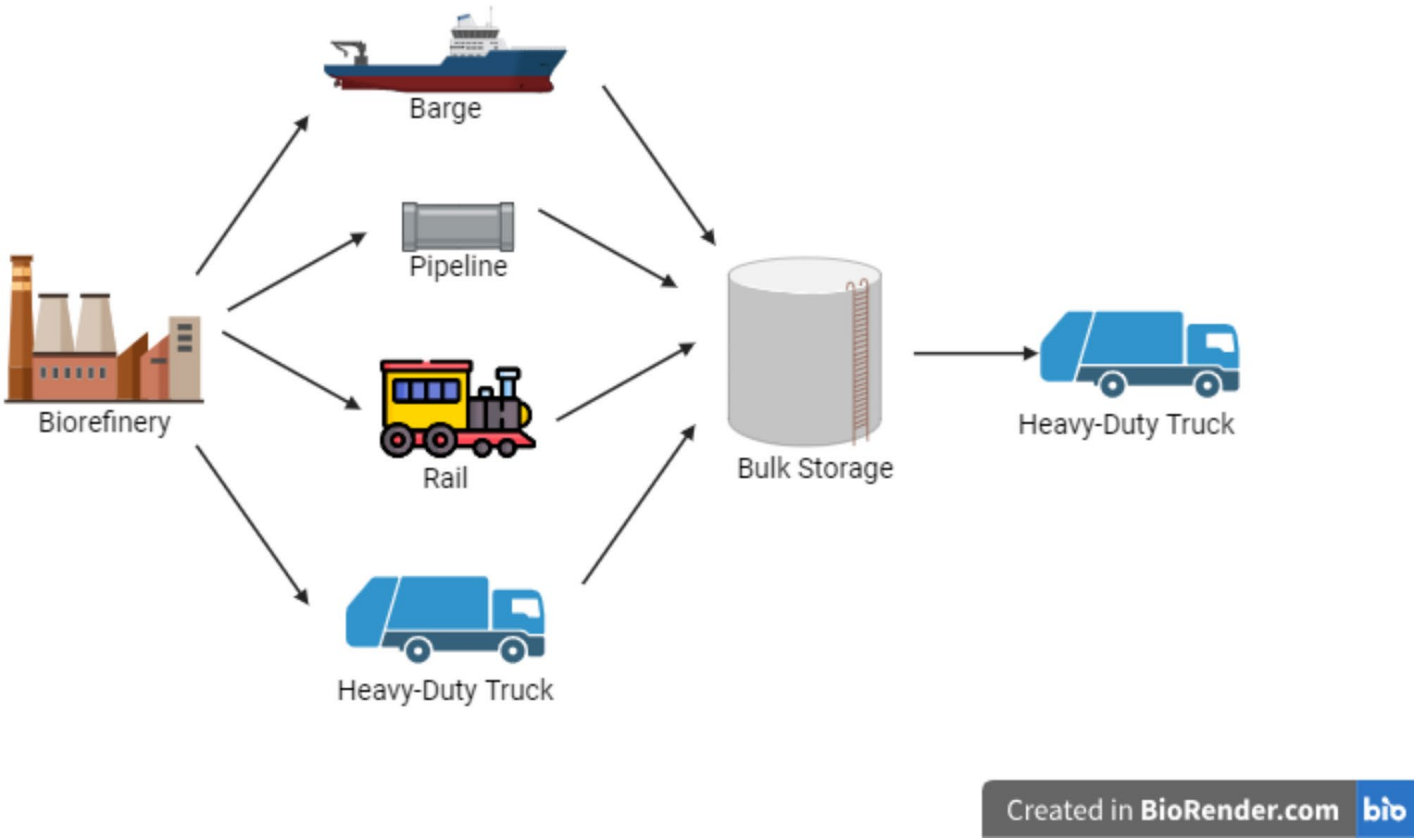
Renewable diesel transportation (Conceptualised by authors and created using BioRender.com).

Following production, the renewable diesel produced is transported to its end-use destination for utilisation in heavy-duty trucks and other transportation sectors (Fig. 4). The transportation stage introduces additional emissions and energy requirements, depending on the distance to distribution centres and end consumers. This phase is critical in determining the “well-to-wheel” emissions, which include all upstream and downstream activities related to renewable diesel use.

The biorefinery pathways analysed in this study were adapted from the GREET^®^ database. However, it was modified to reflect the real-world scenerios. While the fundamental emission factors remained unchanged, adjustments were made to process parameters, energy inputs, and system boundaries based on recent literature and experimental data. This change accounts for regional variations and technological updates in energy sourcing, ensuring that the energy consumption and associated emissions are more accurately represented. This adjustment allows for a more direct comparison of the environmental performance of different biorefinery pathways without introducing external uncertainties. Moreover, a uniform US-based electricity grid has been chosen for all the pathways to maintain uniformity and robustness across the analysis and to be able to provide a global outlook.

### Pathway-I

The process begins with the cultivation of algae sourced from a well (Fig. 3a). This algae biomass is the primary feedstock for the renewable diesel production pathway. The growth stage is critical to determine the overall biomass availability for subsequent processing. Algae is selected as a feedstock due to its high productivity and potential for carbon capture, which positions it as a promising candidate for biofuel production. Once the algae biomass is cultivated, it is transported from the wastewater treatment plant (WWTP) to the HTL biorefinery (Tables S5, S6). This transportation stage introduces energy consumption related to logistics, depending on the distance and transportation methods used. Although the transportation phase is relatively straightforward, its contribution to the life cycle’s carbon footprint must be accounted for, particularly in fuel and electricity use.

Dewatering represents a key step before the algae biomass enters the HTL process. The algae biomass used for HTL has an ash content of 39.3%, affecting biomass’s conversion efficiency into biofuels. Dewatering is necessary to reduce the water content in the algae, making it more suitable for thermal processing (Table S7). During dewatering, electricity is consumed, and the source of this electricity can influence the overall environmental performance. The energy intensity of this step is a critical factor, as it can affect the carbon footprint and energy return on investment of the biofuel production chain.

The HTL process is the core conversion technology that transforms the algae biomass into renewable diesel. HTL utilises a range of inputs, including natural gas, sulfuric acid, and electricity (Table S4). Natural gas, sourced from shale and conventional recovery pathways, is used as a process fuel. Additionally, gaseous hydrogen, generated internally, and water are vital components of the HTL reaction environment. By-products such as nitrogen and phosphoric acid (P_2_O_5_) are also generated, potentially contributing to the overall resource efficiency of the process.

The efficiency of the HTL system depends on the interaction of these various inputs, particularly the energy sources and chemicals involved. The renewable diesel produced in this stage is a high-value biofuel, with a focus on reducing lifecycle greenhouse gas emissions compared to traditional fossil-based fuels.

### Pathway-II

As a critical input in algae growth, CO_2_ is sourced and transported from offsite facilities to the algae growth ponds (Table S10). The transportation and transfer of CO_2_ from offsite locations rely heavily on energy consumption for which electricity is consumed, and the source of this electricity can influence the overall environmental performance (Fig. 2). The choice of electricity source has a significant impact on the overall GHG emissions of the CO_2_ transport stage.

In addition to the energy use, this stage involves infrastructure and logistics that could affect the environmental footprint. Efficient CO_2_ capture, storage, and transportation technologies are vital for minimising the carbon intensity of the algae cultivation phase, ensuring that the renewable diesel pathway maintains a favourable emissions profile. The algae growth and dewatering stage in CAP serves as the biomass cultivation phase (Fig. 3b). Similar to other algae-based biofuel processes, whole algae biomass is the primary output of the algae cultivation process. The algae are grown in ponds, using carbon dioxide and electricity from the U.S. power mix as key inputs (Fig. 2).

Dewatering is required to reduce the water content of the biomass before further processing. The energy consumption for this step is influenced by the technology used and the percentage of water removal required. The dewatering process is energy-intensive and its environmental impact is highly dependent on the source of electricity and the energy efficiency of the technology employed.In the utilised state-of-technology version of this process, several chemical inputs are introduced during algae growth, including ammonium phosphate and diammonium phosphate for nutrient supplementation (Table S9). Other materials, such as steel, concrete, and high-density polyethylene, are also part of the infrastructure required for algae cultivation, influencing the capital cost and embodied energy in the system (Table S11).

The CAP system represents an integrated approach for converting algae biomass into biofuels, primarily renewable diesel while producing valuable co-products. The process incorporates multiple conversion pathways, both thermochemical and biochemical, to maximise the utilisation of algae biomass. The key inputs to the process include natural gas (sourced through both shale and conventional recovery methods) and sulfuric acid (essential for the biochemical reactions involved in the conversion). Additionally, gaseous hydrogen plays a critical role in facilitating these reactions.

The primary output of the CAP system is renewable diesel which serves as a renewable fuel source. Alongside this, the process generates several displaced resources, including ammonia, nitrogen, and calcium nitrate. These displaced resources offer the potential to reduce the environmental burden on other industries by providing alternative materials. Furthermore, the process yields co-products such as ethanol and ammonium phosphate, which contribute to the economic viability of the system.

Once produced, the renewable diesel from the CAP system is transported to distribution centres and end users (Fig. 4). This stage involves additional fuel consumption and GHG emissions, depending on the distance between the production site and the consumer market.

### Pathway-III

The production of renewable diesel from PFAD, a by-product of palm oil refining, represents a key pathway for the development of second-generation biofuels. This process begins with the refining of crude palm oil into refined, bleached, and deodorised palm oil (RBD), where inputs such as phosphoric acid and electricity from the US power mix are utilised. The primary outputs of this stage are RBD palm oil, widely used in various industries, and PFAD, a lower-value by-product that holds significant potential as a feedstock for biofuel production.

The allocation of PFAD as a by-product or co-product of palm oil refining depends on the economic and environmental assessment methodologies applied. In the present analysis, PFAD is classified as a by-product, allocated based on energy content, and directed toward the production of renewable diesel within a second-generation biorefinery (Table S14). Second-generation biofuels are distinct from first-generation fuels in that they utilise non-food biomass or waste streams, thereby reducing competition with agricultural resources and mitigating the risks of food insecurity.

Once extracted, the PFAD undergoes a transportation phase (Table S15). In the biorefinery, PFAD undergoes conversion to renewable diesel through a series of energy-intensive processes. The key inputs required for this conversion include (i) natural gas, sourced from both shale and conventional recovery methods, which provides the necessary thermal energy for various chemical reactions; (ii) gaseous hydrogen (H_2_), which is compressed and used for hydrotreating, a process that removes oxygen atoms from the biofuel molecules, improving fuel quality and stability; (iii) electricity, drawn from the US’ distributed electricity mix (Fig. 2), powers the facility’s infrastructure and machinery (Table S13). The environmental impact of this conversion step is contingent upon the carbon intensity of the electricity used, highlighting the importance of sourcing energy from low-emission grids.

The primary output of this biorefinery process is renewable diesel, a low-carbon alternative to conventional fossil diesel. In addition, co-products such as propane and naphtha are generated. These by-products contribute to the overall resource efficiency of the biorefinery by providing alternative feedstocks for other industrial applications, further reducing waste and enhancing the economic viability of the process. Following production, the renewable diesel is transported to end-user markets. The transportation phase is energy-intensive, with fuel consumption and GHG emissions largely driven by the mode of transport, fuel type, and shipping distances.

## Results and discussion

### Pathway results

#### Pathway-I

**Fig. 5:**
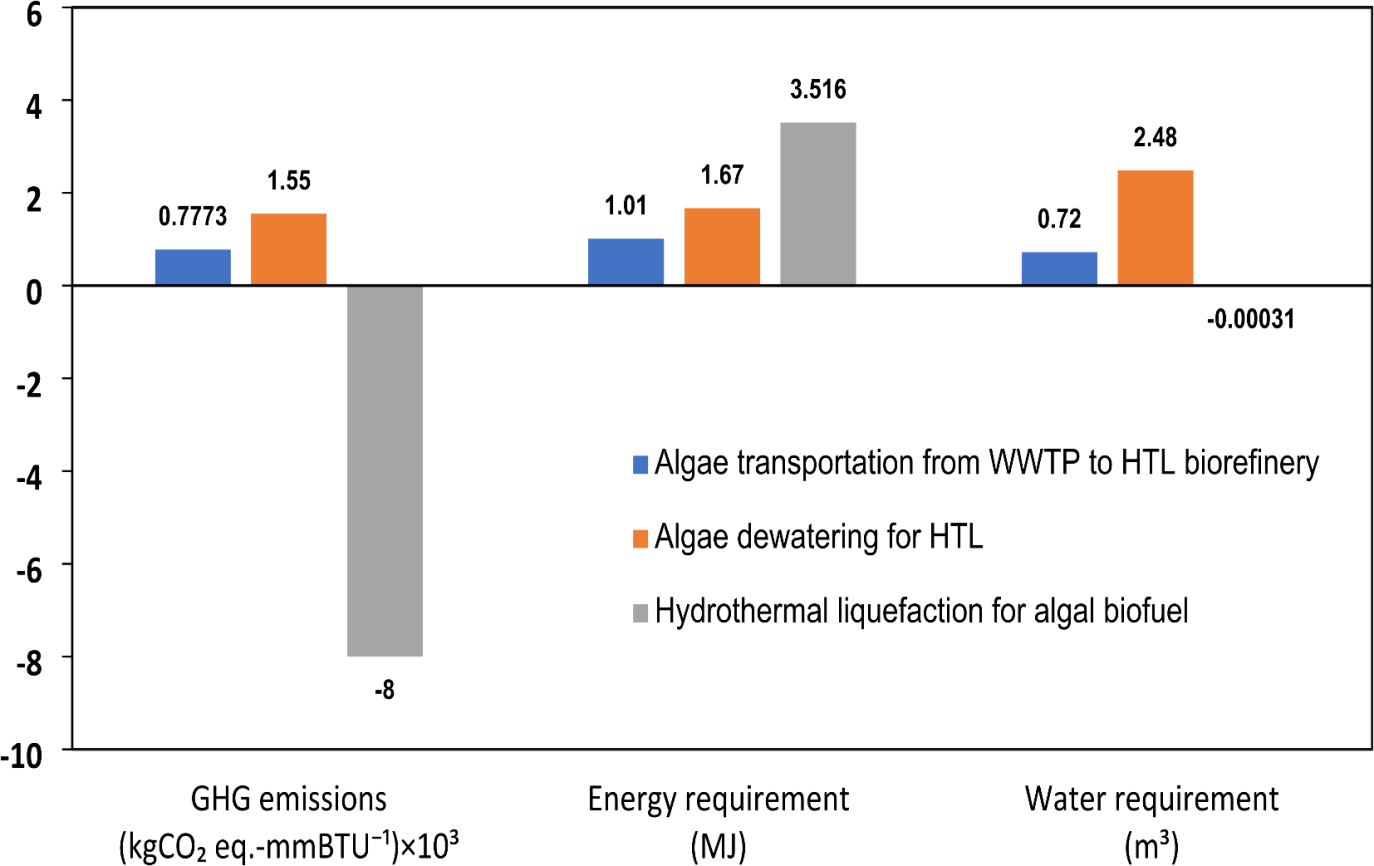
Well-to-product GHG emissions (kg CO_2_-eq. mmBTU^− 1^), Energy requirement (MJ), and Water requirement (m^3^) for Pathway-I.

The findings pertaining to GHG emissions, overall energy utilization, and water footprint associated with the production of 1 mmBTU of renewable diesel via the HTL pathway are depicted in Fig. 5. A comprehensive well-to-product methodology is employed, thereby omitting transportation-related emissions from this particular assessment. The analysis reveals that the HTL process results in a net carbon reduction quantified at −7.99 kg CO_2_-eq for every 1 mmBTU of renewable diesel produced. This negative carbon footprint is ascribed to the intrinsic carbon sequestration potential of algae during the cultivation phase, which effectively mitigates emissions produced during the subsequent processing stages. Nevertheless, it is noted that majority of the emissions correlated with this pathway originate from transportation, thereby highlighting the imperative for enhanced logistical strategies aimed at reducing overall life cycle emissions.

In the context of energy efficiency, HTL demonstrates a significant total energy requirement, approximated at 3715 MJ for each 1 mmBTU of renewable diesel. The considerable energy demand associated with this process is predominantly linked to the dewatering of algae biomass, which constitutes one of the most energy-demanding phases in algae-derived fuel production. Furthermore, the thermal energy necessitated for the liquefaction process itself further intensifies energy consumption, thereby presenting challenges for large-scale implementation, especially in locales where renewable energy sources are not readily accessible.

A notable benefit of the HTL methodology is its distinctive water balance, as it produces more water than it utilizes. The net water generation is quantified at − 0.3247 m^3^ for each 1 mmBTU of renewable diesel, thereby differentiating HTL from traditional biofuel pathways that typically display elevated water footprints. This observation implies that HTL may be particularly advantageous in regions facing water scarcity, where the minimization of water consumption is a crucial determinant of the sustainability of fuel production systems. Additional procedural specifics and input parameters that underpin these conclusions, are outlined in Table S1 of the supplementary materials.

#### Pathway-II

**Fig. 6:**
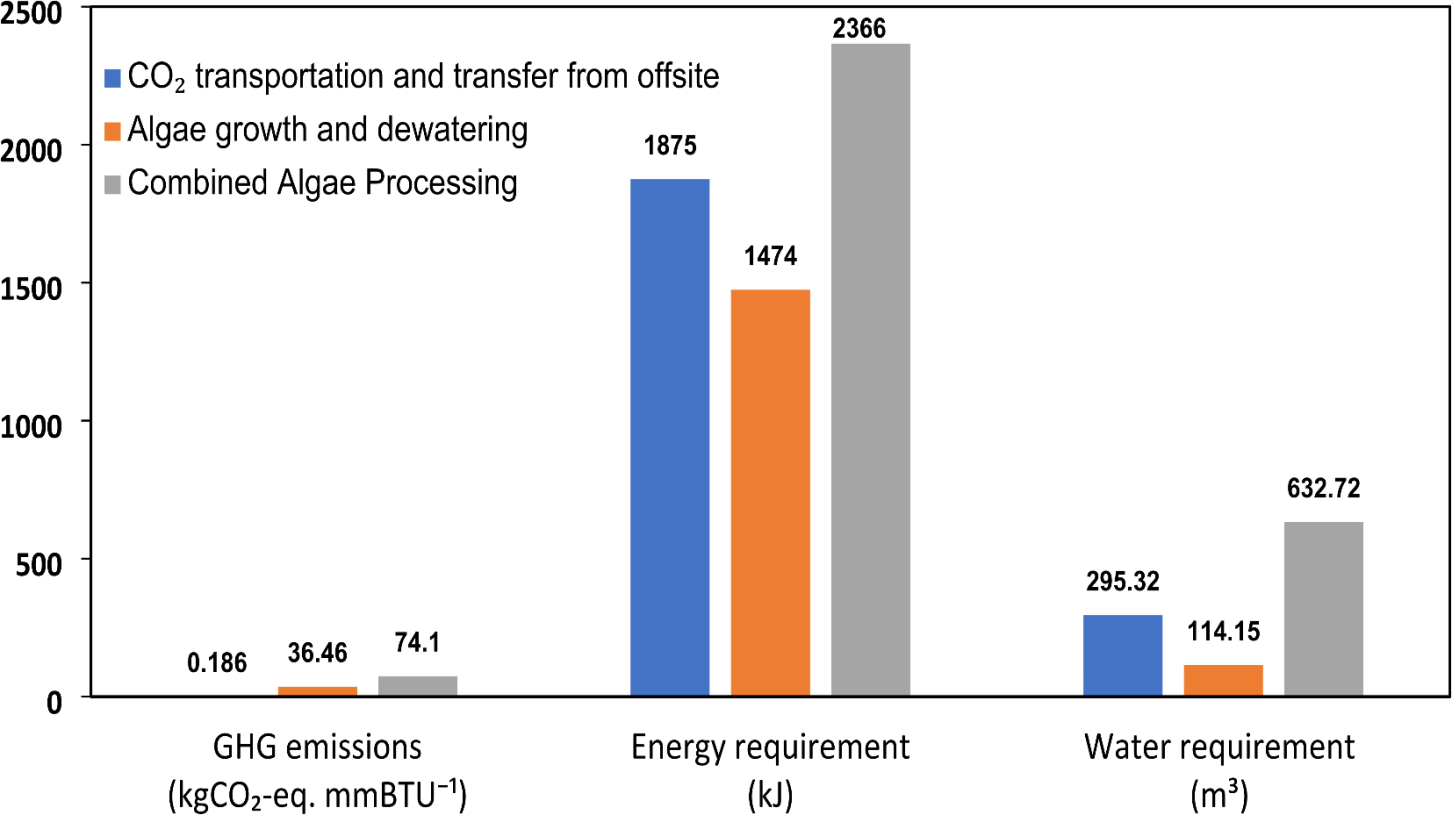
Well-to-product GHG emissions (kg CO_2_-eq. mmBTU^− 1^), Energy requirement (kJ), and Water requirement (m^3^) for Pathway-II.

The environmental and resource utilization metrics for the CAP pathway are illustrated in Fig. 6, providing insights into its greenhouse gas emissions, energy requirements, and water footprint. The CAP pathway generates 78.59 kg CO_2_-eq per 1 mmBTU of renewable diesel, making it the highest emitter among the three pathways evaluated. A significant portion of these emissions, approximately 41.5%, can be attributed to transportation, suggesting that optimizing supply chain logistics may be essential for improving the overall carbon footprint of this pathway. Compared to HTL, CAP does not benefit from substantial carbon sequestration effects, as the integration of biochemical and thermochemical processes in this pathway does not inherently capture and store atmospheric CO_2_ in the same manner as algae cultivation.

The total energy demand for CAP is reported at 2502 MJ per 1 mmBTU of renewable diesel, making it more energy-efficient than HTL. The lower energy consumption in CAP can be linked to its hybrid processing approach, where some biochemical reactions occur at lower temperatures, reducing overall thermal energy requirements. However, despite its relative energy efficiency, the CAP pathway presents notable sustainability challenges due to its high greenhouse gas emissions. The presence of multiple conversion steps and reliance on external sources of CO_2_ introduce additional energy burdens that impact the overall life cycle performance of this pathway.

Unlike HTL, CAP is a net water consumer, with a total water requirement of 0.668 m^3^ per 1 mmBTU of renewable diesel. While this water footprint remains significantly lower than that of the PFAD-based pathway, it still highlights potential limitations, especially in regions where access to freshwater resources is constrained. The infrastructure and chemical inputs required for CAP, including ammonium phosphate and other essential nutrients, contribute to this water demand, as detailed in Table S2.

#### Pathway-III

**Fig. 7:**
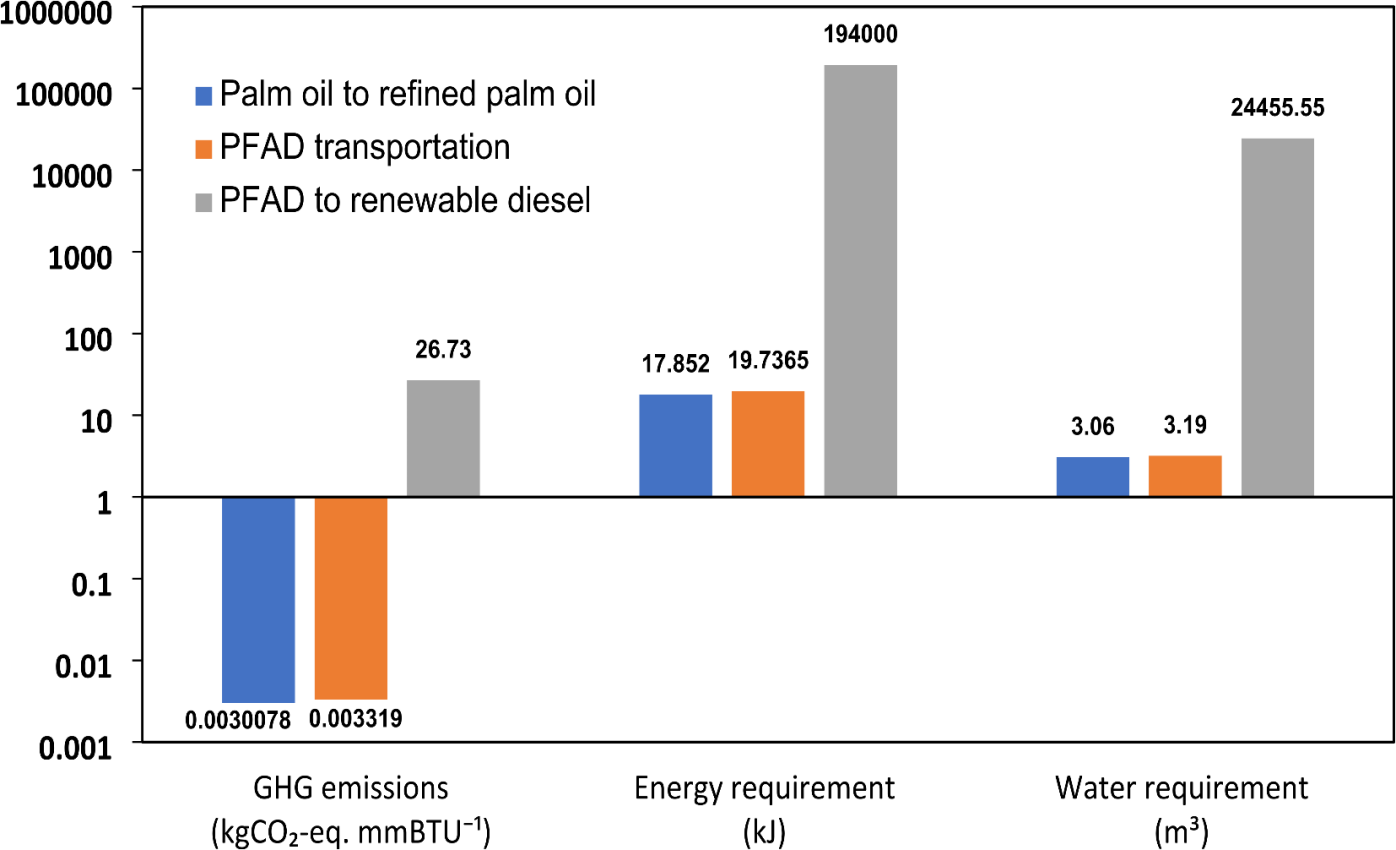
Well-to-product GHG emissions (kg CO_2_-eq. mmBTU^− 1^), Energy requirement (kJ), and Water requirement (m^3^) for Pathway-III (*Note*: Plotted on a logarithmic scale to normalize the values; PFAD: Palm fatty acid distillate).

The results for the PFAD-based pathway, representing a second-generation biorefinery approach, are shown in Fig. 7. This pathway results in GHG emissions of 58.66 kg CO_2_-eq per 1 mmBTU of renewable diesel, a value lower than CAP but significantly higher than HTL. Notably, transportation accounts for 53.33% of these emissions, highlighting the major role of logistics in determining the overall sustainability of PFAD-based diesel. The emissions from feedstock production and refining contribute substantially to the total carbon footprint, as the industrial processing of palm oil introduces additional emissions from electricity and chemical use.

From an energy perspective, the PFAD pathway demonstrates superior efficiency, requiring only 194.03 MJ per 1 mmBTU of renewable diesel. This significantly lower energy requirement compared to HTL and CAP can be attributed to the maturity of hydrotreating technology, which is already widely adopted at an industrial scale. Unlike algae-based pathways, PFAD does not require extensive pre-processing, reducing overall energy burdens and making it a more economically viable option in terms of fuel production costs.

However, the PFAD pathway presents a major sustainability challenge due to its exceptionally high water demand. The total water consumption per 1 mmBTU of renewable diesel is recorded at 24.47 m^3^, far exceeding the requirements of the other pathways. The detailed breakdown of material use for this pathway is provided in Table S3.

#### Comparison of the results

The LCA results of Pathway-I, Pathway-II, and Pathway-III highlight significant differences in environmental performance, energy efficiency, and resource utilisation. Pathway-I demonstrates the most favorable environmental impact, with a net reduction of −7.99 kg CO_2_-eq per 1 mmBTU of renewable diesel produced. This negative carbon footprint can be attributed to the integration of algae-based biomass and hydrothermal liquefaction, which enhances carbon sequestration. Furthermore, Pathway-I exhibits a net positive water balance (−0.3247 m^3^), indicating water generation rather than consumption. However, this environmental benefit is offset by a high total energy requirement of 3715 MJ, which may present challenges for large-scale implementation, particularly in contexts where renewable energy inputs are limited.

Pathway-II demonstrates a lower total energy requirement of 2502 MJ, making it more energy-efficient than Pathway-I. However, this improvement in energy efficiency is accompanied by significantly higher GHG emissions (78.59 kg CO_2_-eq per 1 mmBTU ) and net water requirement of 0.668 m^3^, which may pose sustainability concerns in water-stressed regions. Additionally, 41.5% of the total emissions in Pathway-II are attributed to transportation, underscoring the role of logistical considerations in determining the overall carbon footprint. These findings suggest that while Pathway-II may be a more energy-efficient alternative, its environmental trade-offs must be carefully evaluated.

Pathway-III, a second-generation biorefinery, exhibits substantially higher GHG emissions (58.66 kg CO_2_-eq per 1 mmBTU ) and significantly greater water requirement (24.47 m^3^) compared to the third-generation pathways. While the proportion of emissions from transportation remains comparable at approximately 50%, the absolute values are considerably higher, indicating a less sustainable profile overall. The high water demand associated with Pathway-III is particularly concerning, given that large-scale biofuel production could exacerbate existing water scarcity issues.

The results indicate that third-generation biorefineries offer substantial environmental benefits over second-generation alternatives, with Pathway-I emerging as the most promising in terms of sustainability. However, its high energy demand remains a limiting factor, necessitating further exploration of renewable energy integration to enhance feasibility. Pathway-II provides greater energy efficiency, but its significantly higher GHG emissions and water consumption may hinder its widespread adoption, particularly in regions with stringent environmental regulations. Pathway-III, despite similarities in transportation-related emissions, demonstrates inferior performance compared to third-generation pathways, reinforcing the potential of advanced biorefinery technologies in achieving more sustainable biofuel production. While the findings underscore the environmental advantages of third-generation pathways, large-scale implementation will depend on addressing energy constraints and optimising process efficiencies.

Potential sources of uncertainty include feedstock-specific conversion efficiencies, supply chain logistics, and regional variations in grid emissions. While algae-based biofuels are actively being developed in the US, geographical variations in electricity sources, transport distances, and refining efficiencies may influence LCA results. The model accounts for these factors by incorporating realistic energy and emissions values where data is available. Future work can further refine these estimates by integrating region-specific electricity grids and supply chain logistics to enhance accuracy.

### Evolution of biorefineries: challenges and opportunities

A core objective of this study is to provide a forward-looking introspection into the evolution of biorefineries, highlighting the challenges and opportunities associated with different generations of biofuel production technologies. The pathways examined in this study represent distinct stages in the transition toward more advanced and sustainable biofuel systems. By comparing their environmental performance, energy efficiency, and resource utilization, this study contributes to an understanding of how biorefinery technologies have progressed and where improvements are still required^24,29^.

Second-generation biorefineries, such as PFAD-based diesel production, have gained traction due to their reliance on non-food biomass and industrial by-products rather than edible feedstocks^30^. However, as this study demonstrates, the sustainability of such systems remains contested. The exceptionally high water footprint of PFAD-based diesel (24.47 m^3^ per 1 mmBTU) presents a major concern, particularly in water-scarce regions, while its moderate greenhouse gas emissions (58.66 kg CO_2_-eq per 1 mmBTU) suggest that further optimization is needed^31^. Despite its low energy requirements (194.03 MJ per 1 mmBTU), the overall feasibility of PFAD-based fuels depends on addressing the land-use concerns and emissions associated with palm oil supply chains, as well as improving water efficiency in refining processes^32^.

Third-generation biorefineries, exemplified by HTL and CAP, offer promising pathways for reducing carbon emissions and improving resource efficiency. HTL, in particular, presents a net negative carbon footprint ( 7.99 kg CO_2_-eq per 1 mmBTU) due to algae’s inherent ability to capture and store atmospheric CO_2_, positioning it as a key technology for future carbon-neutral fuel production^25^. Furthermore, the net-positive water balance (−0.3247 m^3^ per 1 mmBTU) observed in this pathway highlights its unique ability to generate water rather than consume it, making it potentially viable in water-scarce regions^33^. However, the major limitation of HTL is its high energy demand (3715 MJ per 1 mmBTU), which could hinder large-scale deployment unless supported by low-carbon electricity sources or process intensification strategies^34^.

CAP, on the other hand, improves energy efficiency but results in substantially higher greenhouse gas emissions (78.59 kg CO_2_-eq per 1 mmBTU), indicating that hybrid biochemical-thermochemical approaches require further refinement to minimize environmental trade-offs^24,35^. While its energy requirement (2502 MJ per 1 mmBTU) is lower than that of HTL, its net water consumption (0.668 m^3^ per 1 mmBTU) suggests that improvements in water recovery and recycling processes will be necessary for long-term feasibility^36^.

This comparative analysis underscores that the evolution of biorefineries is characterized by continuous improvements in feedstock selection, process efficiency, and environmental performance, yet persistent challenges remain^32,34^. Future advancements in bioprocessing efficiency, renewable energy integration, and carbon capture strategies will be critical in determining the scalability and real-world impact of these technologies^37^.

## Conclusions and future research perspective

First-generation biorefineries face challenges, such as land use changes and competition with food production, but second-generation biorefineries (utilising lignocellulosic biomass) offer more sustainable options limited by technical challenges. Third-generation biorefineries, which harness microalgae and CO_2_, hold the highest potential for carbon neutrality, though scalability remains a barrier. Environmental impacts, including emissions, energy consumption, and water use, decrease progressively across biorefinery generations.

As a future research scope, biorefineries have a significant potential for innovation and sustainability. For example, marine biorefineries are emerging as a promising area within this field. Furthermore, given the escalating scarcity of freshwater resources, particularly in arid and semi-arid regions, future biorefineries must explore alternatives, such as the utilisation of seawater as a reaction medium. This strategy is especially viable for large-scale biorefineries situated in coastal regions, thereby aligning with the global sustainability imperative^36^.

Additionally, the exploitation of marine biomass represents a distinctive opportunity to address global energy demands sustainably. By harnessing a broad spectrum of marine organisms, including microalgae and macroalgae, marine biorefineries can contribute to the sustainable production of biofuels and high-value bioproducts, thereby advancing the global energy transition^37–40^.

Technological advancements are poised to play a critical role in the evolution of marine biorefineries. Innovations in cell manufacturing, lipidomic analysis, and nanotechnology-guided lipid extraction are anticipated to enhance the economic viability of large-scale production. Furthermore, developing emerging green technologies, including hydrothermal treatments, advanced fermentation processes, and the deployment of green solvents, is essential for optimising the efficiency and environmental performance of marine biorefineries^33,41^. Future research should focus on the environmental impact and economic feasibility of biorefineries. This includes LCA and the development of integrated models that combine biotechnological and chemical processes.

## Electronic supplementary material

Below is the link to the electronic supplementary material.

Supplementary Material 1

## Data Availability

Relevant data used for, and generated through this study is provided as Supplementary material.
