# Supplementary material for: Life-cycle assessment of three biorefinery pathways across different generations
Source: Sci Rep. 2025 Apr 16;15:13135. doi: 10.1038/s41598-025-96474-w (PMC12003876; doi:10.1038/s41598-025-96474-w)
Supplement: Supplementary file 1 — Supplementary Material 1 [file 41598_2025_96474_MOESM1_ESM.docx]

**Supplementary Information**

**Table of abbreviations**

| CO_2_ | Carbon dioxide | CAP | Combined algae processing |
| --- | --- | --- | --- |
| GHG | Greenhouse gas | mmBTU | One million British thermal units |
| GREET^®^ | Greenhouse gases, regulated emissions, and energy use in transportation | PFAD | Palm fatty acid distillate |
| HTL | Hydrothermal liquefaction | RBD | Refined, bleached, and deodorized |
| LCA | Life cycle assessment | US | United States |
| LCSA | Life cycle sustainability assessment | WWTP | Wastewater treatment plant |
| MJ | Megajoule |  |  |

For the purpose of recapitulation, this paper provides the LCI data used, which are obtained from previously published literature, government reports, and some enterprise real-world data.

1. **Emission Values**

The emissions generated for the three pathways (HTL, CAP, PFAD) have been provided in **Tables S1-S3**.

**Table S1.** Well-to-wheel emissions for hydrothermal liquefaction pathway

| Well-to-wheel emissions | Units | Algae transportation  from WWTP to HTL biorefinery | Algae dewatering  for HTL | HTL for algal biofuel | Renewable diesel  transportation | Sum of all pathway results |
| --- | --- | --- | --- | --- | --- | --- |
| VOC | kg | 1.145×10^-7^ | 2.185×10^-7^ | -2.72×10^-5^ | -2.75×10^-2^ | -0.03 |
| CO | kg | 1.579×10^-6^ | 2.709×10^-6^ | -2.56×10^-5^ | -2.64×10^-2^ | -0.03 |
| NO_x_ | kg | 1.083×10^-6^ | 1.9791×10^-3^ | -6.14×10^-5^ | -6.35×10^-2^ | -0.06 |
| PM_10_ | kg | 6.086×10^-8^ | 1.294×10^-7^ | -1.68×10^-5^ | -1.77×10^-2^ | -0.02 |
| SO_x_ | kg | 4.720×10^-8^ | 2.486×10^-7^ | -1.48×10^-4^ | -1.56×10^-1^ | -0.16 |
| CH_4_ | kg | 9.226×10^-8^ | 2.054×10^-8^ | -8.12×10^-5^ | -8.52×10^-2^ | -0.09 |
| N_2_O | kg | 2.977×10^-9^ | 1.002×10^-5^ | -4.47×10^-6^ | -4.70×10^-3^ | 0.00 |
| CO_2_ | kg | 7.462×10^-4^ | 1.481×10^-5^ | -4.20×10^-2^ | -4.04×10^0^ | -4.04 |
| GHG | kg | 7.773×10^-4^ | 1.550×10^-4^ | -8.00×10^-3^ | -7.99×10^0^ | -7.99 |

**Table S2.** Well-to-wheel emissions for combined algae processing pathway

| Well-to-wheel emissions | Units | CO_2_ transportation  and transfer from offsite | Algae growth and dewatering | CAP | Renewable Diesel  transportation | Sum of all pathway results |
| --- | --- | --- | --- | --- | --- | --- |
| VOC | kg | 1.516×10⁻⁵ | 9.104×10⁻⁶ | 2.202×10⁻⁵ | 2.442×10⁻² | 0.02 |
| CO | kg | 5.547×10⁻⁵ | 1.830×10⁻⁵ | 5.387×10⁻⁵ | 5.744×10⁻² | 0.06 |
| NO_x_ | kg | 1.340×10⁻⁴ | 2.836×10⁻⁵ | 6.955×10⁻⁵ | 7.470×10⁻² | 0.07 |
| PM_10_ | kg | 2.820×10⁻⁵ | 5.205×10⁻⁶ | 6.074×10⁻⁶ | 6.450×10⁻³ | 0.01 |
| SO_x_ | kg | 1.771×10⁻⁴ | 3.332×10⁻⁵ | 4.148×10⁻⁵ | 4.380×10⁻² | 0.04 |
| CH_4_ | kg | 2.846×10⁻⁴ | 8.087×10⁻⁵ | 2.235×10⁻⁴ | 2.360×10⁻¹ | 0.24 |
| N_2_O | kg | 4.085×10⁻⁶ | 7.378×10⁻⁷ | 3.690×10⁻⁶ | 3.900×10⁻³ | 0.00 |
| CO_2_ | kg | 1.763×10⁻¹ | 3.379×10⁻² | 6.627×10⁻³ | 7.031×10¹ | 70.53 |
| GHG | kg | 1.86×10⁻¹ | 2.601×10¹ | 2.619×10¹ | 3.929×10¹ | 78.59 |

**Table S3.** Well-to-wheel emissions for palm fatty acid distillation pathway

| Well-to-wheel emissions | Units | Palm oil to refined palm oil | PFAD transportation | PFAD to renewable diesel | Renewable Diesel  transportation | Sum of all pathway results |
| --- | --- | --- | --- | --- | --- | --- |
| VOC | kg | 1.496×10⁻⁷ | 2.433×10⁻⁷ | 2.017×10⁻³ | 5.042×10⁻³ | 7.06×10⁻³ |
| CO | kg | 4.545×10⁻⁷ | 7.527×10⁻⁷ | 6.004×10⁻³ | 1.072×10⁻² | 1.67×10⁻² |
| NO_x_ | kg | 1.952×10⁻⁶ | 4.103×10⁻⁶ | 1.089×10⁻² | 5.659×10⁻² | 6.75×10⁻² |
| PM_10_ | kg | 3.311×10⁻⁷ | 5.181×10⁻⁷ | 9.376×10⁻⁴ | 4.881×10⁻³ | 5.82×10⁻³ |
| SO_x_ | kg | 3.717×10⁻⁶ | 5.172×10⁻⁶ | 7.439×10⁻³ | 3.846×10⁻² | 4.59×10⁻² |
| CH_4_ | kg | 1.724×10⁻⁶ | 1.892×10⁻⁶ | 3.373×10⁻² | 3.649×10⁻² | 7.02×10⁻² |
| N_2_O | kg | 2.935×10⁻⁸ | 3.173×10⁻⁸ | 2.655×10⁻⁴ | 3.178×10⁻⁴ | 5.80×10⁻⁴ |
| CO_2_ | kg | 1.398×10⁻³ | 1.542×10⁻³ | 1.138×10¹ | 1.379×10¹ | 2.51×10¹ |
| GHG | kg | 3.079×10⁻³ | 3.319×10⁻³ | 2.673×10¹ | 3.192×10¹ | 5.86×10¹ |

**2. Inventory analysis**

The inventory parameters for each step of the three renewable diesel pathways (HTL, CAP, PFAD) have been provided in sections 2.1-2.3 as **Tables S4-S16** [1-13]. For the process of renewable diesel transportation, the authors have developed a custom transportation route (described in section 2 of the manuscript) data, which has been kept same for all three pathways, for which has been provided in **Tables** **S8**, **S12** and **S17**.

**2.1. Hydrothermal liquefaction pathway (Pathway-I)**

The inventory parameters over the process of renewable diesel production in the HTL (Pathway-I) are shown in **Tables S4-S8** [1-5].

**Table S4.** Inventory data for HTL (refining) process

| Category |  | Parameter | Unit | Quantity |
| --- | --- | --- | --- | --- |
|  |  | Natural gas | mmBTU | 0.15980 |
| Input | Energy and Power | Electricity | mmBTu | 3.33114 |
|  | Materials | Whole algae biomass | kg | 106.4856 |
|  |  | Sulfuric acid | kg | 9.980214 |
|  |  | Mo/Ni/Al_2_O_3_ spent catalyst | g | 37.81872 |
|  |  | Water | L | 245.8562 |
|  |  | Sodium chloride | kg | 4.095516 |
|  |  | Magnesium oxide | kg | 4.100731 |
| Output | By-products | Nitrogen | kg | 2.34465 |
|  |  | Phosphoric acid | kg | 11.8752 |
|  |  | Ash/solid residue | kg | 41.86 |
|  | Production | Renewable diesel | mmBTU | 1 |

**Table S5.** Inventory data for the algae growth phase taking place in WWTP

| Category |  | Parameter | Unit | Quantity |
| --- | --- | --- | --- | --- |
|  |  | Natural gas | mmBTU | - |
| Input  Output | Energy and Power | Electricity | mmBTu | - |
|  | Materials | Whole algae biomass | kg | 1 |
|  | Production | Whole algae biomass | kg | 1 |

**Table S6.** Inventory data for the transportation of algae biomass from WWTP to HTL biorefinery using heavy-duty truck

| Category |  | Parameter | Unit | Quantity |
| --- | --- | --- | --- | --- |
|  | Energy and Power | Electricity | Wh | - |
|  | Materials | - | - | .- |
|  | Transport | Distance | km | 80 |

**Table S7.** Inventory data for algae dewatering for HTL

| Category |  | Parameter | Unit | Quantity |
| --- | --- | --- | --- | --- |
| Input | Energy and Power | Electricity | Wh | 12.4885 |
|  | Materials | Whole algae biomass | kg | 1.6487 |
| Output | Production | Dewatered algae | kg | 1 |
|  | By-products | Water | kg | 0.6847 |

**Table S8.** Inventory data for the transportation of renewable diesel for use in heavy duty trucks

| Category |  | Parameter | Unit | Quantity |
| --- | --- | --- | --- | --- |
|  | Energy and Power | Electricity | Wh | - |
|  | Materials | - | - | .- |
|  | Transport | Distance | km | 150 |

**2.2. Combined algae processing (Pathway-II)**

The inventory parameters over the process of renewable diesel production in the CAP pathway (Pathway-II) are shown in **Tables S9-S8** [4, 6-9].

**Table S9.** Inventory data for combined algae processing (refining) process

| Category |  | Parameter | Unit | Quantity |
| --- | --- | --- | --- | --- |
|  |  | Natural gas | mmBTU | 0.000454 |
| Input | Energy and Power | Electricity | mmBTU | 2.34114 |
|  | Materials | Whole algae biomass | kg | 28.48231 |
|  |  | Sulfuric acid | kg | 4.462332 |
|  |  | Gaseous hydrogen | kg | 12.95112 |
|  |  | Corn steep liquor | kg | 7.886610 |
|  |  | Ammonia | kg | 15.49123 |
|  |  | N-hexane | kg | 2.870000 |
|  |  | Ethanol | kg | 1.141000 |
|  |  | Diammonium phosphate | g | 95.3457 |
|  |  | Nafion dry polymer | kg | 4.68821 |
|  |  | Water | kg | 668.0010 |
|  |  | Magnesium oxide | kg | 4.100731 |
| Output | By-products | Nitrogen | kg | 2.34465 |
|  |  | Ammonia | kg | 5.76470 |
|  |  | Calcium nitrate | g | 58.8737 |
|  |  | Diammonium Phosphate | kg | 4.61180 |
|  | Production | Renewable diesel | mmBTU | 1 |

**Table S10.** Inventory data for the CO_2_ transfer from offsite

| Category |  | Parameter | Unit | Quantity |
| --- | --- | --- | --- | --- |
|  |  | Natural gas | BTU | 34.9865 |
| Input | Energy and Power | Electricity | BTu | 652.7112 |
|  | Materials | Carbon dioxide | kg | 1 |

**Table S11.** Inventory data for algae growth and dewatering phase

| Category |  | Parameter | Unit | Quantity |
| --- | --- | --- | --- | --- |
|  | Energy and Power | Electricity | kWh | 0.6031 |
| Input | Materials | Whole algae biomass | kg | 1 |
|  |  | Carbon dioxide | kg | 2.2253 |
|  |  | Ammonia | g | 20.0341 |
|  |  | Diammonium phosphate | g | 9.67371 |
|  |  | Concrete | g | 24.1987 |
|  |  | Steel | g | 0.96760 |
|  |  | Irom | g | 3.38661 |
|  |  | High density polymer | g | 5.41850 |
| Output | By-products | - | - | - |
|  | Production | Whole algae biomass | Kg | 1 |

**Table S12.** Inventory data for the transportation of renewable diesel for use in heavy duty trucks

| Category |  | Parameter | Unit | Quantity |
| --- | --- | --- | --- | --- |
|  | Energy and Power | Electricity | Wh | - |
|  | Materials | - | - | .- |
|  | Transport | Distance | km | 150 |

**2.3. Palm fatty acid distillation pathway (Pathway-II)**

The inventory parameters over the process of renewable diesel production in the PFAD pathway (Pathway-III) are shown in **Tables S13-S16** [10-13].

**Table S13.** Inventory for palm fatty acid distillate to renewable diesel

| Category |  | Parameter | Unit | Quantity |
| --- | --- | --- | --- | --- |
|  |  | Natural gas | mmBTU | 441.72524 |
|  | Energy and Power | Hydrogen | mmBTU | 1951.8092 |
| Input |  | Electricity | mmBTU | 154.090446 |
|  | Materials | PFAD | kg | 36.99 |
| Output | By-products | Propane | kg | 0.0207 |
|  |  | Naphtha | kg | 0.002 |
|  | Production | Renewable diesel | kg | 0.45 |

**Table S14.** Inventory for refined palm oil production with PFAD as a by-product

| Category |  | Parameter | Unit | Quantity |
| --- | --- | --- | --- | --- |
|  | Energy and Power | Residual crude oil | BTU | 195.8083 |
| Input |  | Electricity | BTU | 17.64396 |
|  | Materials | Phosphoric Acid | g | 0.4309 |
| Output | By-products | PFAD | kg | 0.0204 |
|  | Production | Refined palm oil | Kg | 0.4331 |

**Table S15.** Inventory data for the transportation of PFAD to biorefinery

| Category |  | Parameter | Unit | Quantity |
| --- | --- | --- | --- | --- |
|  | Energy and Power | Electricity | Wh | - |
|  | Materials | - | - | .- |
|  | Transport | Distance | km | 77 |

**Table S16.** Inventory data for the transportation of renewable diesel for use in heavy duty trucks

| Category |  | Parameter | Unit | Quantity |
| --- | --- | --- | --- | --- |
|  | Energy and Power | Electricity | Wh | - |
|  | Materials | - | - | .- |
|  | Transport | Distance | km | 150 |

**References**

1. Zhu, Y., Schmidt, A. J., Valdez, P. J., Snowden-Swan, L. J., Edmundson, S. J. (2022). *Hydrothermal Liquefaction and Upgrading of Wastewater-Grown Microalgae: 2021 State of Technology* (No. PNNL-32695). Pacific Northwest National Lab.(PNNL), Richland, WA (United States).
2. Tan, E. C. D., Talmadge, M., Dutta, A., Hensley, J., Schaidle, J., Biddy, M., Humbird, D., et al. (2015). *Process Design and Economics for the Conversion of Lignocellulosic Biomass to Hydrocarbons via Indirect Liquefaction.* National Renewable Energy Lab. (NREL), Golden, CO (United States).
3. Snowden-Swan, L., Li, S., Jian, Y., Thorson, M., Schmidt, A., Seiple, T., Billing, J., Santosa, M., Hart, T., Fox, S., Cronin, D., Ramasamy, K., Anderson, D., Hallen, R., Norton, J., and Fonoll, A. X. (2022). *Wet Waste Hydrothermal Liquefaction and Biocrude Upgrading to Hydrocarbon Fuels: 2021 State of Technology*. PNNL-32731. Pacific Northwest National Laboratory, Richland, WA.
4. Cai, H., Ou, L., Wang, M., Davis, R., Dutta, A., Harris, K., and Li, S. (2022). *Supply Chain Sustainability Analysis of Renewable Hydrocarbon Fuels via Indirect Liquefaction, Hydrothermal Liquefaction, Combined Algal Processing, and Biochemical Conversion: Update of the 2021 State-of-Technology Cases* (No. ANL/ESD-22/5). Argonne National Lab. (ANL), Argonne, IL (United States).
5. Ovsyannikova, E., Kruse, A., Becker, G. C. Feedstock-dependent phosphate recovery in a pilot-scale hydrothermal liquefaction bio-crude production. *Energies*, ***13*(2),** 379 (2020).
6. D’Souza, S., Johnston, J., Thomas, V. M., Harris, K., Tan, E. C., Chance, R. R., Yuan, Y. (2024). Integrating direct air capture with algal biofuel production to reduce cost, energy, and GHG emissions. *Journal of CO2 Utilization*, *86*, 102911.
7. Wiatrowski, M., Davis, R. (2023). *Algal Biomass Conversion to Fuels via Combined Algae Processing (CAP): 2022 State of Technology and Future Research* (No. NREL/TP-5100-85662). National Renewable Energy Laboratory (NREL), Golden, CO (United States).
8. Davis, R., Markham, J., Kinchin, C., Grundl, N., Tan, E. C., Humbird, D. (2016). *Process design and economics for the production of algal biomass: algal biomass production in open pond systems and processing through dewatering for downstream conversion* (No. NREL/TP-5100-64772). National Renewable Energy Lab. (NREL), Golden, CO (United States).
9. Dong, T., Knoshaug, E. P., Davis, R., Laurens, L. M., Van Wychen, S., Pienkos, P. T., Nagle, N. (2016). Combined algal processing: A novel integrated biorefinery process to produce algal biofuels and bioproducts. *Algal Research*, *19*, 316-323.
10. Honorato de Oliveira, B. F., de França, L. F., Fernandes Corrêa, N. C., Ribeiro, N. F. D. P., Velasquez, M. (2021). Renewable diesel production from palm fatty acids distillate (Pfad) via deoxygenation reactions. Catalysts, 11(9), 1088.
11. Pehnelt, G., Vietze, C. (2013). Recalculating GHG emissions saving of palm oil biodiesel. *Environment, Development and Sustainability*, *15*, 429-479.
12. Kalnes, T. N., Koers, K. P., Marker, T., Shonnard, D. R. (2009). A techno-economic and environmental life cycle comparison of green diesel to biodiesel and syndiesel. *Environmental Progress Sustainable Energy: An Official Publication of the American Institute of Chemical Engineers*, *28*(1), 111-120.
13. Xu, H., Lee, U., Wang, M. (2020). Life-cycle energy use and greenhouse gas emissions of palm fatty acid distillate derived renewable diesel. *Renewable and Sustainable Energy Reviews*, *134*, 110144.
